# Characteristics of smear-positive pulmonary tuberculosis with normal chest radiographic findings during a tuberculosis outbreak in northeast Malaysia: A cross-sectional study

**DOI:** 10.51866/oa.595

**Published:** 2024-09-24

**Authors:** Abdul Muin Mohd Redhuan, Mohd. Yusof Abu Bakar, Ahmad Fuat Mohd Shahriman, Amri Nur Syafiqa, Amirudin Noor Faizah, Wan Mohd Zohdi Wan Wahida, Ibrahim Idora, Ahmad Arfah, Muhammad Irham Nordeena, Ahmad Abdul Jalil, Abdul Majid Jasmaniah, Ibrahim Mohd Zukri bin

**Affiliations:** 1 MB BCh BAO, Dr Fam Med, Klinik Kesihatan Simpang Kuala, Jalan Kuala Kedah, Alor Setar, Kedah, Malaysia. Email: redhuanmuin@gmail.com; 2 MD, MMed (Fam Med), Klinik Kesihatan Lubuk Merbau, Jalan Pejabat, Padang Terap, Kedah, Malaysia.; 3 MD, MMed (Fam Med), Klinik Kesihatan Sungai Tiang, Pendang, Kedah, Malaysia.; 4 MBBS, MMed (Fam Med), Klinik Kesihatan Malau, Baling, Kedah, Malaysia.; 5 MBBS, MMed (Fam Med), Klinik Kesihatan Tunjang, Jitra, Kedah, Malaysia.; 6 MBBS, MMed (Fam Med), Klinik Kesihatan Kepala Batas, Jitra, Kedah, Malaysia.; 7 MD, MMed (Fam Med), Klinik Kesihatan Ayer Hitam, Ayer Hitam, Kedah, Malaysia.; 8 MBBS, MMed (Fam Med), Klinik Kesihatan Changlun, Changlun, Kedah, Malaysia.; 9 MB BCh BAO, MMed (Fam Med), Klinik Kesihatan Kodiang, Kodiang, Kedah, Malaysia.; 10 MD, MMed (Fam Med), Klinik Kesihatan Tunjang, Jitra, Kedah, Malaysia.; 11 MD, MMed (Rad), Jabatan Radiologi, Hospital Sultanah Bahiyah, Jalan Langgar, Alor Setar, Kedah, Malaysia.; 12 MD, MPH (Occp. Health), Pejabat Kesihatan Daerah Kulim, Jalan Hospital, Kulim, Kedah, Malaysia.

**Keywords:** Tuberculosis, Disease outbreak, Pulmonary tuberculosis, Thoracic radiography

## Abstract

**Introduction:**

Tuberculosis (TB) remains a highly prevalent disease in Malaysia. Early identification using cost-effective methods such as chest radiography can reduce the health burden of a TB epidemic. However, normal chest radiographic findings are common. This study aimed to evaluate the characteristics of smear-positive pulmonary TB with normal chest radiographic findings during an outbreak.

**Methods:**

A cross-sectional study was conducted by reviewing the medical records of 56 pulmonary TB cases registered at Kodiang Health Clinic from April to October 2022. Smear-negative and extrapulmonary TB cases were excluded. Relevant information was extracted from the medical records and recorded in a case report form for data management and analysis.

**Results:**

Approximately 60.7% of the cases had symptoms lasting >2 weeks, and 89.3% showed abnormal findings upon clinical examination. Additionally, 73.2% had sputum acid-fast bacilli counts of ≥1+, and the sputum *Mycobacterium tuberculosis* culture and sensitivity test findings were positive in 82.1% of the cases. The proportion of smear-positive pulmonary TB with normal chest radiographic findings was 42.9%. The factors associated with smear-positive pulmonary TB with normal chest radiographic findings included being under 18 years old (P=0.021), being a student (P=0.010), being single (P=0.012) and being asymptomatic (P=0.04).

**Conclusion:**

Normal chest radiographic findings may lead to a misdiagnosis of smear-positive pulmonary TB, especially during an outbreak. Therefore, active case detection among close contacts with risk factors for normal radiographic findings should consider additional supportive tests.

## Introduction

Tuberculosis (TB) continues to pose a significant health challenge globally, including in Malaysia.^1^ Despite rigorous public health measures to eliminate TB in Malaysia, there is no sign of it slowing down.^2^ The multifaceted nature of TB makes its management and eradication complex, especially when considering the various environmental, social and human factors contributing to its persistence.^3^ The diversity of clinical presentations associated with TB complicates its diagnosis, as it can manifest in different ways, making it challenging to identify and treat promptly.^4^ The diagnostic challenge emphasises the need for improved screening methods and increased awareness among healthcare providers and the general population.

Chest radiography remains the primary modality for the assessment of TB, especially in high-burden areas.^5^ It is considered a safe and effective modality because of its high sensitivity, wide availability and relatively low cost.^6^ Despite its good sensitivity and specificity in diagnosing TB, normal chest radiographic findings in pulmonary TB are common. This would often then lead to missed or delayed diagnoses.^7^ Typical chest radiographic findings in TB are not found especially among populations with human immunodeficiency virus (HIV) infection and concomitant extrapulmonary TB.^8^ Normal chest radiographic findings are also more common among asymptomatic cases, which are usually identified through active case detection via contact tracing.^8,9^

The Kodiang locality in Kedah was affected by a TB outbreak in April 2022. A TB outbreak is defined by the Centers for Disease Control and Prevention as the presence of more TB cases than expected within a geographic area in a particular period and evidence of recent transmission of *Mycobacterium tuberculosis* (MTB) among such cases.^10^ Initial examinations in the 2022 outbreak revealed normal chest radiographic findings regardless of the symptoms. Therefore, we aimed to study the proportion and characteristics of smear-positive pulmonary tuberculosis (PTB) with normal chest radiographic findings during an outbreak.

## Methods

This cross-sectional study involved a medical record review of PTB cases registered at Kodiang Health Clinic during a TB outbreak in 2022. All new TB cases registered in the TB registry from April to October 2022 were identified, and cases that fulfilled the criteria were selected and analysed. All newly diagnosed and registered smear-positive PTB cases were included. All of these cases were diagnosed based on positive findings from a sputum acid-fast bacilli (AFB) smear, a *Mycobacterium tuberculosis* culture and sensitivity (MTB C&S) test or an Xpert MTB/ RIF assay. Conversely, smear-negative PTB, non-tuberculosis mycobacterium (NTM), extrapulmonary TB and TB cases registered before April 2022 but still under treatment and follow-up were excluded from the study. The sample size was not calculated because all cases detected throughout the outbreak period were included in the study.

The first posteroanterior chest radiograph taken during TB evaluation was used for the analysis and sent for reporting by the radiologist in the tertiary hospital. The radiologist reviewed all chest radiographs as part of routine clinical care and generated written reports. The chest radiographs were graded as no lesion, minimal, moderate or severe based on the Malaysian Clinical Practice Guideline for the Management of Tuberculosis.^11^ Relevant information was extracted from the medical records and recorded in a case report form for data management; such information included the sociodemographic data, associated factors, symptoms, clinical presentations and investigation findings.

Some of the clinical symptoms of PTB include cough, fever, appetite loss, weight loss, haemoptysis and fatigue. The presence of at least one symptom is considered to indicate a symptomatic case. The symptoms are categorised by duration as lasting less than 2 weeks or more. Abnormal physical examination findings include lung consolidation or effusion (e.g. rales, crepitations or bronchial breath sounds), swollen or tender lymph nodes or clubbing.

The data obtained were analysed using the Statistical Package for the Social Sciences version 26 (Armonk, NY: IBM Corp). The categorical data were presented as frequencies and percentages. The association between various factors and smear-positive PTB with normal chest radiographic findings was determined using simple logistic regression. A P-value of <0.05 was considered statistically significant.

## Results

A total of 62 medical records were reviewed for study eligibility. Four cases of smear-negative PTB and two cases of extrapulmonary TB were excluded from the study. Thus, a total of 56 medical records of smear-positive PTB cases were analysed. The mean age of the patients was 37 years. Table 1 summarises the sociodemographic characteristics of the patients.

**Table 1:** Sociodemographic characteristics (N=56).

Variable	n (%)
***Age, year***	
≤18 years	16 (28.6)
19-59 years	31 (55.4)
≥60 years	9 (16.1)
***Sex***	
Male	26 (46.4)
Female	30 (53.6)
***Race***	
Malay	52 (92.9)
Chinese	3 (5.4)
Indian	0(0)
Others	1 (1.8)
***Occupation***	
Unemployed	15(26.8)
Employed	23(41.1)
Sindcni	18(32.1)
***Educational level***	
No Formal education	6(10.7)
Primary/secondary education	45 (80.4)
Tertiary education	5 (8.9)
***Marital status***	
Single	23(41.1)
Married	32(57.1)
Divorced	1 (1.8)

The proportion of patients with normal chest radiographic findings was 42.9%. The majority of the patients presented with clinical signs and symptoms of PTB, with 60.7% exhibiting symptoms for >2 weeks and 89.3% showing abnormal findings upon clinical examination. The sputum MTB C&S test findings were positive in 82.1% (Table 2), and no NTM was detected. The analysis of the risk factors for contracting PTB (Table 3) revealed that 64.3% of the cases were close contacts and that none of the cases were HIV-positive. Approximately 92.9% of the patients completed at least two primary doses of SARS-CoV-2 vaccination. Those who were aged under 18 years (P=0.01), single (P=0.012), students (P=0.01) and asymptomatic (P=0.04) were more likely to have smear-positive PTB with normal chest radiographic findings (Table 4).

**Table 2:** Clinical presentation, sputum analysis and chest radiographic findings (N=56).

Variable	n (%)
***Symptom duration***	
Asymptomatic	15 (26.8)
Symptoms lasting <2 weeks	7 (12.5)
Symptoms lasting ≥2 weeks	34 (60.7)
***Clinical examination finding***	
Normal	6 (10.7)
Abnormal	50 (89.3)
***Chest radiographic finding***	
Normal	24 (42.9)
Mild	27 (48.2)
Moderate	5 (8.9)
Severe	0 (0)
***Sputum MTB C&S test finding***	
Positive	46 (82.1)
Negative	10 (17.9)
***Sputum AFB smear finding***	
Scanty	15 (26.8)
1+	36 (64.3)
2+	3 (5.4)
3+	2 (3.5)

**Table 3:** Risk factors for smear-positive PTB (N=56).

Variable	n (%)
***Close contact***	
Yes	36 (64.3)
No	26 (35.7)
***Diabetes mellitus***	
Yes	12(21.4)
No	44 (78.6)
***HIV***	
Yes	0(0)
No	56(100)
***Substatue use***	
Yes	0(0)
No	56(100)
***Current smolter***	
Yes	12(21.4)
No	44 (78.6)
***History of SARS-Co V-2 infection***	
Yes	20 (35.7)
No	36 (64.3)
***SARS-Co V-2 vaccination***	
0 doses	4(7.1)
1 dose	0 (0.0)
2 doses	36 (64.3)
3 doses	16 (28.6)
***History of PTB***	
Yes	3 (5.4)
No	53 (94.6)

**Table 4:** Factors associated with smear-positive PTB with normal chest radiographic findings.

Variable	Normal chest radiographic finding n=24 (%)	Abnormal chest radiographic finding n=32 (%)	Crude OddsRatio (OR)	95% CI	P-value
*Age*					
≤18 years	11 (68.8)	5 (31.2)	5.341	1.262-16.917	0.021^*^
19-59 years	10 (32.2)	21 (67.7)	1		
≥60 years	3 (33.3)	6 (66.7)	0.004	0.217-5.084	0.953
***Sex***					
Male	14 (53.8)	12 (67.7)	1		
Female	10 (33.3)	20 (66.7)	2.333	0.791-6.885	0.125
***Race***					
Malay	23 (44.2)	29 (55.8)	2.379	0.232-24.415	0.466
Others	1 (25.0)	3 (75.0)	1		
***Occupation***					
Unemployed	4 (26.7)	11 (73.3)	0.831	0.195-3.538	0.831
Employed	7 (30.4)	16 (69.6)	1		
Students	13 (72.2)	5 (27.8)	5.943	1.524-23.181	0.010^*^
***Educational level***					
No formal education	2 (33.3)	4 (66.7)	1		
Primary/secondary education	20 (44.4)	25 (55.6)	1.666	0.265-9.644	0.608
Tertiary education	2 (40.0)	3 (60.0)	1.333	0.113-15.704	0.819
***Marital status***					
Single	15 (62.5)	9 (37.5)	4.259	1.375-13.184	0.012^*^
Married	9 (28.1)	23 (71.9)	1		
***Symptom duration***					
Asymptomatic	10 (66.7)	5 (33.3)	12.000	1.118-128.836	0.04^*^
Symptoms lasting <2 weeks	1 (14.3)	6 (85.7)	1		
Symptoms lasting >2 weeks	13 (38.2)	21 (61.8)	3.714	0.401-34.443	0.248
***Close contact***					
Yes	15 (41.7)	21 (58.3)	1		
No	9 (45.0)	11 (55.0)	1.145	0.380-3.449	0.809
***Diabetes mellitus***					
Yes	4 (33.3)	8 (66.7)	1		
No	20 (45.5)	24 (54.5)	1.667	0.437-6.358	0.455
***Current smoker***					
Yes	5 (41.7)	7 (58.3)	1		
No	19 (43.2)	25 (56.8)	1.067	0.292-3.878	0.925
***History of SARS-CoV-2 infection***					
Yes	7 (35.0)	13 (65.0)	1.662	0.538-5.134	0.378
No	17 (47.2)	19 (52.8)	1		
***SARS-CoV-2 vaccination***					
0 doses	2 (8.3)	2 (6.2)	1		
2 doses	21 (87.5)	15 (46.9)	1.400	0.177-11.083	0.750
3 doses	1 (4.2)	15 (46.9)	0.067	0.004-1.116	0.067
***Sputum MTB C&S test finding***					
Positive	18 (75.0)	28 (87.5)	1		
Negative	6 (25.0)	4 (12.5)	2.333	0.577-9.432	0.234
***Sputum AFB smear finding***					
Scanty	8 (33.3)	7 (21.9)	4.571	0.409-51.138	0.217
1+	15 (62.5)	21 (65.6)	2.857	0.290-28.194	0.369
≥2+	1 (4.2)	4 (12.5)	1		

* Statistically significant at P<0.05

## Discussion

Our study reported a larger proportion of smear-positive PTB cases with normal chest radiographic findings than did the national registry (9.9%),^12^ studies in Western countries (4.8%–18%) and studies in other Asian countries (2.5%-9.9%).8,9,^13-15^ However, this study is the only study evaluating the phenomenon in an outbreak with differing epidemiological contexts and levels of TB transmission. Moreover, the disparity is due to the variation in the study populations and diagnostic criteria. Previous studies concentrated on specific populations, such as those with advanced HIV-related immunosuppression. They also included only bacteriologically confirmed PTB cases based on the MTB C&S test findings, while our study included all cases with initial sputum AFB-positive results. This is because the long waiting time for the MTB C&S test findings necessitates prompt diagnosis in outbreak management to ensure effective disease control.

Our findings revealed that the patients who were aged 18 years and below and were students were five to six times more likely to have normal chest radiographic findings. This is consistent with previous reports indicating that Bacillus Calmette-Guerin (BCG) vaccination among children and adolescents could provide protection, lessen disease severity and restore chest radiographic findings.^16,17^ BCG vaccination has been included in the Malaysian vaccination progra mme since 1961 and is routinely given to newborns at birth.^18^

In the present study, the single patients were four times more likely to have normal chest radiographic findings. Spouses of patients with smear-positive TB are at a higher risk of contracting PTB due to prolonged and close exposure, as they live together, share a sleeping room and frequently nurse the patient.^19^ Close contact with infectious PTB cases results in a higher bacterial burden, leading to more severe chest radiographic changes.^20^ Our findings are consistent in showing that close contact was not significantly associated with normal chest radiographic findings.

Similar to previous reports,^8,21^ the current study showed that the asymptomatic patients exhibited a 12-fold higher likelihood of presenting normal chest radiographic findings. This phenomenon may be attributed to rigorous early screening within the community, thereby enhancing the detection rate of PTB cases among asymptomatic individuals. Early detection results in a lower bacterial load among asymptomatic patients, potentially explaining the normal findings on chest radiographs.^22^ However, our findings indicated that the sputum AFB count was not associated with normal chest radiographic findings. The limited number of sputum samples with high AFB loads (≥2+) in our study sample may have constrained our ability to identify significant effects.

Herein, we found no significant correlation between SARS-CoV-2 vaccination and smear-positive PTB among the patients with normal chest radiographic findings. To our knowledge, no prior studies have investigated the impact of SARS-CoV-2 vaccination on the chest radiographs of patients with PTB. However, previous research has indicated that SARS-CoV-2 vaccination offers some protection against active TB.^23^ Our study was conducted during the COVID-19 pandemic, while vaccination programmes, including third-dose vaccinations, were ongoing in Malaysia. Despite a high vaccination rate in our study sample (92.5% receiving at least two primary doses), the small number of unvaccinated individuals may have limited our ability to detect significant effects.

To the best of our knowledge, this study is the first study to be conducted in Malaysia to investigate the relationship between various factors and smear-positive PTB with normal chest radiographic findings during an outbreak. For instance, we studied the association between SARS-CoV-2 vaccination and smear-positive PTB with normal chest radiographic findings, a topic that remains novel during the endemic phase. Despite these strengths, our study has the following limitations: The data were collected through retrospective medical record reviews, and we did not clarify the symptoms and perform physical examinations among the patients. Therefore, there could be under- or over-reporting of the sociodemographic data, clinical presentations and risk factors for PTB. Another limitation is that our sample size was small, which may render some findings statistically insignificant.

## Conclusion

In conclusion, the proportion of smear-positive PTB with normal chest radiographic findings is high during an outbreak. The risk factors associated with this condition are being under 18 years old, being a student, being single and being asymptomatic. Therefore, active case detection and screening of PTB, especially among close contacts with such risk factors, may need to consider additional supportive tests to rule out active PTB.

## Data sharing statement

The data that support the findings of this study are available on request from the corresponding author. These data are not publicly available since they contain information that could compromise the privacy of the participants.

**How does this paper make a difference in general practice?**

- Understanding the characteristics of patients with pulmonary tuberculosis who are more likely to have normal chest radiographic findings allows practitioners to identify patients at risk of being misdiagnosed.
- Standard protocols for using chest radiography alone in asymptomatic close contacts should consider other supportive investigations such as the sputum acid-fast bacilli test or Xpert MTB/RIF assay.
- Effective active case detection during a tuberculosis epidemic or outbreak allows effective disease control to prevent catastrophic outcomes.
